# Interaction of smoking and metabolic syndrome in increasing the recurrence risk of colorectal cancer in a Chinese male cohort: a retrospective study

**DOI:** 10.1038/s41598-018-19322-0

**Published:** 2018-01-17

**Authors:** Da-Zhi Chen, Fei-yang Ji, Qiao-Mai Xu, Xiao-Xin Wu, Chao Cai, Ling-Jian Zhang, Lan-Juan Li

**Affiliations:** 1 State Key Laboratory for Diagnosis and Treatment of Infectious Diseases, Collaborative Innovation Center for Diagnosis and Treatment of Infectious Diseases, the First Affiliated Hospital, College of Medicine, Zhejiang University, 310003 Hangzhou, China; 2 Department of Infection and Liver Diseases, Liver Research Center, The First Affiliated Hospital of Wenzhou Medical University, No. 2 Fuxue Lane, Wenzhou, 325000 China

## Abstract

Whether smoking and metabolic syndrome (MetS) can affect colorectal carcinoma (CRC) prognosis remains debatable. Therefore, the present study aimed to examine the individual and combined effects of smoking and MetS on the prognosis of patients with localized CRC, including stage I to III disease. The relationship among smoking status, MetS, and CRC was assessed in 838 Chinese male patients. Cox proportional hazards regression analysis was used to evaluate CRC prognosis adjusted for clinicopathological variables. Relative excess risk of interaction (RERI), attributable proportion (AP), and synergy index (SI) were used to evaluate additive interactions between smoking and MetS. The presence of MetS was an independent risk factor for low rates of recurrence-free survival (RFS) but not for overall survival (OS). However, smoking was independently associated with both poor RFS and OS. Furthermore, the recurrence risk for current smokers with MetS was 1.62 times as high as the sum of risks in patients exposed to each risk factor alone. In conclusion, current smoking habit is a risk factor for both recurrence and cancer-specific mortality in CRC patients, while MetS is an independent predictor for CRC recurrence. Furthermore, these two factors have an additive effect on the recurrence risk of CRC.

## Introduction

As one of the most prevalent cancers in the world, colorectal carcinoma (CRC) has dramatically increased in incidence in Asia over the past few decades^1^. Additionally, CRC is one of the leading causes of cancer-related death and results in low quality of life in survivors worldwide, including in China^2,3^. According to a survey in the USA, patients with non-metastatic CRC have five-year survival rates of approximately 69.2–90.1%^4^. However, these survivors are continuously at risk of recurrence and other long-term sequelae, which may influence survival rates. As revealed by some studies, an appropriate postoperative management can significantly improve prognosis^5^. Thus, it is critical to identify risk factors related to adverse outcomes in CRC patients and adopt appropriate strategies.

In previous studies, smoking has been shown to lead to the development of CRC^6,7^. As noted by several studies, smoking is significantly related to mortality and recurrence of CRC^8,9^. Metabolic syndrome (MetS) is a group of diseases consisting of different metabolic derangements (such as central obesity, hypertension, hyperglycemia, and dyslipidemia) with debatable association with CRC. Previous studies have demonstrated that MetS not only increases the risk of developing CRC but also results in poor prognoses^10,11^. Nonetheless, other studies supported that MetS may has no apparent effect on CRC outcomes^12^. Although several studies have investigated the association among smoking status, MetS and CRC, no study has concentrated on the combined effect of smoking and MetS on CRC prognosis. Moreover, there are fewer female smokers than are male smokers. Thus, the present study aims to investigate the individual and combined effect of smoking and MetS on CRC prognosis in Chinese male patients to complement this field of research.

## Results

### General characteristics of the study participants

Among the 838 study subjects, the number of never, former, and current smokers was 320 (38.2%), 431 (51.4%), and 87 (10.4%), respectively. Additionally, the number of subjects with and without MetS was 215 (25.7%) and 623 (74.3%), respectively. Furthermore, the number of never, former, and current smokers without MetS was 254 (30.3%), 306 (36.5%), and 63 (7.5%) respectively, while the number of never, former, and current smokers with MetS were 66 (7.9%), 125 (14.9%) and 24 (2.9%), respectively. Body mass index (BMI), systolic blood pressure (SBP), diastolic blood pressure (DBP), triglycerides (TGs) and fasting glucose levels were significantly higher among participants with MetS with different smoking statuses than in corresponding participants without MetS (Table 1). In addition, current smoker CRC patients with MetS were more likely to develop recurrence that were other CRC patients.

**Table 1:** Characteristics of participants by Metabolic Syndrome and Smoking Status.

Characteristics	Non-Metabolic Syndrome (n = 623)			Metabolic Syndrome (n = 215)			P value
	Never smoker	Former smoker	Current smoker	Never smoker	Former smoker	Current smoker	
Total number	254	306	63	66	125	24	
Age (years)	50.12 ± 10.45	51.52 ± 12.61	49.72 ± 14.06	49.76 ± 11.64	52.61 ± 12.49	51.76 ± 12.64	0.321
BMI (kg/m^2^)	22.81 ± 4.19	23.86 ± 4.32	22.66 ± 3.91	24.96 ± 3.51	25.72 ± 4.12	25.89 ± 4.02	0.001*
SBP (mmHg)	117.96 ± 21.18	121.16 ± 23.43	119.96 ± 20.82	134.18 ± 19.9	134.19 ± 20.3	136.52 ± 20.5	0.001*
DBP (mmHg)	75.16 ± 10.28	74.83 ± 10.31	74.13 ± 9.23	78.13 ± 8.32	77.23 ± 10.21	80.42 ± 9.52	0.001*
Triglycerides (mmol/L)	1.54 ± 1.87	1.62 ± 1.71	1.49 ± 1.55	2.21 ± 1.55	2.52 ± 1.81	2.82 ± 1.65	0.009*
HDL (mmol/L)	1.21 ± 0.325	1.29 ± 0.341	1.32 ± 0.481	1.31 ± 0.421	1.33 ± 0.382	1.41 ± 0.421	0.212
LDL (mmol/L)	2.51 ± 1.21	2.62 ± 1.18	2.57 ± 0.98	2.49 ± 0.91	2.49 ± 0.99	2.52 ± 0.81	0.651
Fasting Glucose (mmol/L)	5.02 ± 3.21	5.11 ± 2.32	5.21 ± 2.92	5.42 ± 3.42	5.51 ± 3.21	5.72 ± 2.92	0.013*
CEA (ng/ml)	21.5 ± 100.3	22.8 ± 101.4	23.5 ± 89.9	23.1 ± 92.3	22.1 ± 100.4	24.1 ± 99.5	0.731
Stage							0.390
I	48 (18.9)	68 (22.2)	10 (15.9)	11 (16.7)	22 (17.6)	3 (12.5)	
II	109 (42.9)	137 (45.1)	26 (41.2)	24 (36.4)	47 (37.6)	9 (37.5)	
III	97 (38.2)	101 (32.7)	27 (42.9)	31 (46.9)	56 (44.8)	12 (50.0)	
Location							0.846
Ascending, transverse and descending	68 (26.8)	72 (23.5)	15 (23.8)	23 (34.8)	35 (28.0)	6 (25.0)	
Sigmoid	72 (28.3)	92 (30.1)	18 (28.6)	18 (27.3)	32 (25.6)	5 (20.8)	
Rectum	114 (44.8)	142 (46.4)	30 (47.6)	25 (37.9)	58 (46.4)	13 (54.2)	
Differentiation							0.607
Well/Moderate differentiated	209 (82.2)	263 (85.9)	48 (79.4)	57 (86.4)	102 (81.6)	18 (75.0)	
Pooly/Undifferentiated	45 (17.8)	43 (14.1)	15 (20.6)	9 (13.6)	23 (18.4)	6 (25.0)	
Metformin treatment	41 (16.1)	44 (14.4)	12 (19.0)	22 (33.3)	55 (44.0)	12 (50.0)	0.001*
Statin treatment	39 (15.4)	42 (13.7)	9 (14.3)	21 (31.8)	49 (39.2)	8 (33.3)	0.001*
Cancer-specific mortality	53 (20.8)	71 (23.2)	22 (34.9)	12 (18.2)	25 (20.0)	8 (33.3)	0.121
Recurrence	32 (12.6)	51 (16.7)	20 (31.7)	19 (28.9)	37 (29.6)	16 (66.7)	0.001*
Metabolic Syndrome component ^a^							
I	24 (9.4)	21 (6.9)	7 (11.1)	54 (81.8)	105 (84.0)	17 (70.8)	0.001*
II	138 (54.3)	170 (55.6)	34 (54.0)	52 (78.8)	100 (80.0)	21 (87.5)	0.001*
III	102 (40.2)	126 (41.2)	24 (38.1)	54 (81.2)	105 (84.0)	17 (70.8)	0.001*
IV	78 (31.0)	99 (32.4)	22 (34.9)	57 (86.4)	101 (80.8)	21 (87.5)	0.001*

Abbreviations: BMI, body mass index; SBP, systolic blood pressure; DBP, diastolic blood pressure; HDL, high density lipoprotein; LDL low density lipoprotein.

Note: Data are expressed as mean ± standard deviation and n (%); *represent the P value ≤ 0.05 for chi-square test or ANOVA test. ^a^I, BMI ≥ 25 kg/m^2^; II, anti-hypertensive drug administration and (or) SPB ≥ 140 mmHg or DPB ≥ 90 mmHg; III, TG ≥ 1.7 mmol/L and (or) HDL < 0.9 mmol/L (male), <1.0 mmol/L (female); IV, FPG ≥ 6.1 mmol/L or 2 h postprandial glucose ≥ 7.8 mmol/L.

### Cox analysis of risk factors related to overall survival and recurrence-free survival

The mean follow-up duration was 40.6 ± 20.1 months. The rates of cancer-specific mortality and recurrence were 191 (22.7%) and 175 (20.8%), respectively. In univariate Cox analysis, the levels of high-density lipoprotein (HDL) cholesterol, low-density lipoprotein (LDL) cholesterol, and CEA, tumor stage and differentiation, metformin treatment and smoking status were significant predictive factors of overall survival (OS) in CRC patients. In the multivariate Cox analysis, differentiation, metformin treatment, tumor stage, and smoking status were still independent factors associated with OS after adjustment for HDL, LDL, and CEA levels and stage (Table 2). Additionally, HDL and CEA levels, tumor stage and differentiation, metformin treatment, MetS, and smoking status were significant predictive factors for recurrence-free survival (RFS), as determined by univariate analysis. However, only tumor stage, metformin treatment, MetS, and smoking status were significantly related to RFS after adjustment for the above risk factors in multiple analyses (Table 3).

**Table 2:** Cox proportional hazards regression models of risk factors associated with Overall Survival.

Characteristics	Univariable			Multivariable		
	HR	95%CI	P value	HR	95%CI	P value
Age (years)	1.01	0.99–1.04	0.540			
BMI (kg/m^2^)	0.96	0.81–1.10	0.231			
SBP (mmHg)	1.12	0.91–1.34	0.451			
DBP (mmHg)	0.92	0.81–1.04	0. 531			
Triglycerides (mmol/L)	0.95	0.81–1.12	0.612			
HDL (mmol/L)	1.21	1.09–1.71	0.019*	1.21	0.95–1.51	0.231
LDL (mmol/L)	1.31	1.17–1.53	0.021*	1.29	0.91–1.99	0.313
Fasting Glucose (mmol/L)	1.01	0.93–1.09	0.711			
CEA (ng/ml)	1.10	1.02–1.19	0.030*	1.11	0.89–1.21	0.198
Stage	1.31	1.02–2.01	0.002*	1.52	1.21–2.34	0.001*
I	1.00			1.00		
II	1.02	0.79–1.02	0.312	1.12	0.86–1.24	0.392
III	2.81	1.89–3.98	0.001*	2.41	1.61–3.21	0.001*
Location	1.21	0.81–1.53	0.324			
Ascending, transverse and descending	1.00					
Sigmoid	1.12	0.91–1.21	0.721			
Rectum	1.32	0.82–1.51	0.623			
Differentiation	0.82	0.71–0.92	0.005*	0.86	0.71–0.97	0.029*
Metformin treatment	0.91	0.79–0.97	0.011*	0.89	0.75–0.94	0.022 *
Statin treatment	0.97	0.89–1.032	0.121			
MetS	1.21	0.72–1.53	0.417			
Smoking status	2.61	1.23–4.12	0.005*	2.98	1.33–4.96	0.001*
Never smoker	1.00			1.00		
Former smoker	1.03	0.80–1.12	0.512	1.22	0.91–1.41	0.191
Current smoker	2.89	1.21–3.87	0.001*	3.35	1.46–5.68	0.001*

Abbreviations: BMI, body mass index; SBP, systolic blood pressure; DBP, diastolic blood pressure; HDL, high density lipoprotein; LDL, low density lipoprotein.

Note: *represent the P value ≤ 0.05.

**Table 3:** Cox proportional hazards regression models of risk factors associated with Recurrence-free Survival.

Characteristics	Univariable			Multivariable		
	HR	95%CI	P value	HR	95%CI	P value
Age (years)	1.10	0.98–1.04	0.401			
BMI (kg/m^2^)	1.16	0.82–1.29	0.313			
SBP (mmHg)	1.10	0.93–1.33	0.514			
DBP (mmHg)	1.20	0.92–1.44	0. 614			
Triglycerides (mmol/L)	1.01	0.86–1.15	0.492			
HDL (mmol/L)	1.12	1.00–1.21	0.030*	1.31	0.91–1.61	0.489
LDL (mmol/L)	1.33	0.97–1.54	0.241			
Fasting Glucose (mmol/L)	1.11	0.94–1.29	0.589			
CEA (ng/ml)	1.12	1.02–1.29	0.021*	1.12	0.94–1.26	0.361
Stag	1.28	1.09–1.93	0.013*	1.41	1.21–1.98	0.005*
I	1.00			1.00		
II	1.09	0.89–1.13	0.151	1.21	0.92–1.43	0.179
III	2.11	1.22–2.81	0.001*	1.91	1.31–2.89	0.001*
Location	1.29	0.94–1.56	0.432			
Ascending, transverse and descending	1.00					
Sigmoid	1.21	0.89–1.22	0.692			
Rectum	1.13	0.93–1.33	0.521			
Differentiation	0.89	0.81–0.98	0.010*	1.00	0.88–1.18	0.182
Metformin treatment	0.89	0.74–0.94	0.023*	0.82	0.72–0.91	0.015 *
Statin treatment	1.02	0.92–1.221	0.224			
MetS	2.12	1.25–3.02	0.014*	2.11	1.23–2.81	0.025*
Smoking status	2.75	1.33–4.22	0.010*	3.45	1.51–5.02	0.001*
Never smoker	1.00			1.00		
Former smoker	1.10	0.98–1.33	0.327	1.11	0.92–1.31	0.291
Current smoker	2.99	1.29–3.98	0.001*	3.62	1.67–5. 15	0.006*

Abbreviations: BMI, body mass index; SBP, systolic blood pressure; DBP, diastolic blood pressure; HDL, high density lipoprotein; LDL, low density lipoprotein.

Note: *represent the P value ≤ 0.05.

Table 4 shows the association of individual components of MetS with OS and RFS. In univariate analysis, elevated blood pressure (BP) and hyperglycemia exerted a negative effect on OS and RFS, while dyslipidemia had a positive effect on OS and RFS. Furthermore, multivariate analysis demonstrated similar results after adjustment for age, CEA, stage, tumor location, differentiation, metformin treatment, statin treatment and smoking status.

**Table 4:** Cox proportional hazards regression models of Metabolic syndrome components associated with Overall and Recurrence-free survival among colorectal cancer patients.

Metabolic syndrome component	Overall Survival						Disease-free Survival					
	Univariate			Multivariable			Univariate			Multivariable		
	HR	95%CI	P value	HR	95%CI	P value	HR	95%CI	P value	HR	95%CI	P value
I	1.24	0.91–1.61	0.321	1.13	0.93–1.06	0.218	1.18	0.96–1.32	0.227	1.02	0.92–1.03	0.167
II	1.22	1.06–1.34	0.018*	1.12	1.00–1.24	0.021*	1.19	1.03–1.32	0.014*	1.29	1.04–1.51	0.010*
III	0.78	0.66–0.91	0.015*	0.60	0.53–0.84	0.013*	0.91	0.80–0.97	0.028*	0.83	0.75–0.93	0.035*
IV	1.23	1.11–1.52	0.041*	1.13	1.06–1.48	0.032*	1.29	1.12–1.51	0.017*	1.32	1.09–1.64	0.011*

Note: I, BMI ≥ 25 kg/m^2^; II, anti–hypertensive drug administration and (or) SPB ≥ 140 mmHg or DPB ≥ 90 mmHg; III, TG ≥ 1.7 mmol/L and (or) HDL < 0.9 mmol/L (male), < 1.0 mmol/L (female); IV, FPG ≥ 6.1 mmol/L or 2 h postprandial glucose ≥ 7.8 mmol/L. When perform multivariable analysis, each component was adjusted with age, CEA, stage, location, differentiation and smoking status. ^a^Multivariate cox an^a^lysis was adjusted with age, CEA, stage, location, differentiation, metformin treatment, statin treatment and smoking status. *Represent the P value < 0.05.

### Combined effect of smoking and metabolic syndrome on recurrence

**Figure 1:**
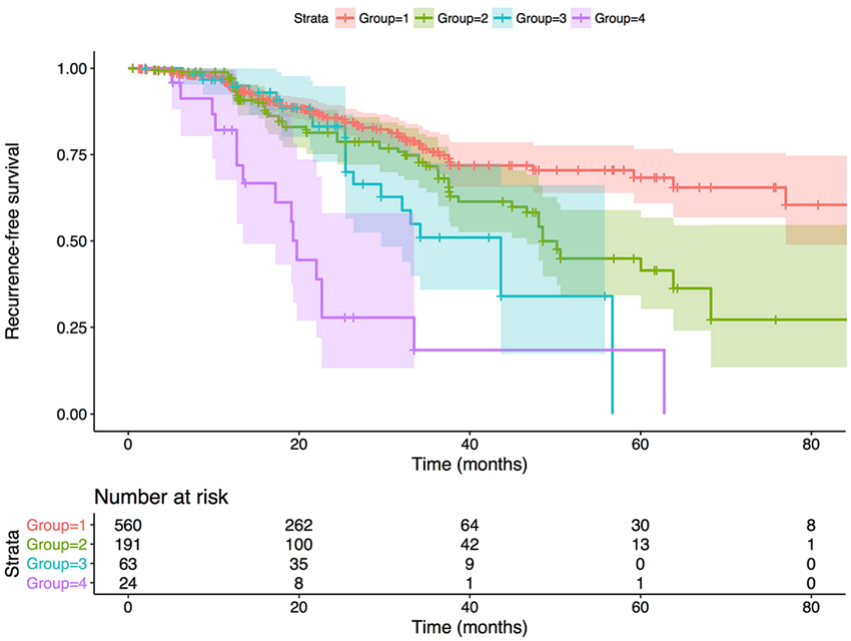
Kaplan-Meier plot showing the recurrence-free survival in patients stratified by MetS and smoking status over the follow-period (p for log-rank test < 0.001). Group 1: non-MetS with non-smoker; Group 2: MetS with non-smoker; Group 3: non-MetS with current smoker; Group 4 MetS with current smoker. The light color shade surrounding each curve indicates 95% CI.

Consistent with previous results, hazard ratios (HRs) were not different between “never” and “former” smokers in either univariate or multivariate Cox analyses for OS and RFS (Tables 2 and 3). Thus, we categorized “never” and “former” smokers into the non-smoker group. As displayed in Fig. 1, RFS rates were significantly higher in patients without MetS or smoking history or neither than in patients who had MetS and who were smokers during the follow-up period (P < 0.001). Furthermore, the patients were divided into four subgroups according to their MetS and smoking status (Table 5). Compared with non-smokers without MetS, the HRs of recurrence were 2.08 (95% confidence interval (CI): 1.11–3.89), 3.39 (95% CI: 2.20–5.20), and 6.62 (95% CI: 4.03–10.87) for non-smokers with MetS, current smokers without MetS, and current smokers with MetS, respectively. Thus, there was a significant additive effect of MetS and smoking status on recurrence after adjustment for age, CEA, stage, tumor location, differentiation, metformin treatment and statin treatment. The value of relative excess risk due to interaction (RERI) was 2.16 (95% CI: 0.67–3.64). In other words, there were 2.16 relatively excess risks due to the additive interaction. Furthermore, the attributable proportion (AP) due to interaction was 0.33 (95% CI: 0.18–0.47), indicating that 33.0% of recurrence due to both risk factors was attributable to the additive interaction. Finally, the synergy index (SI) was 1.62 (95% CI: 1.22–2.15), suggesting that the risk of recurrence in current smokers with MetS was 1.62 times as high as the sum of risks in the participants exposed to each risk factor alone.

**Table 5:** Interaction analysis between Metabolic syndrome and smoking status on recurrence.

Metabolic Syndrome	Smoking status	Case	Total Number	HR (95%CI)	P value
No	Non-smoker	81 (14.5)	560	1.0	
Yes	Non-smoker	56 (29.3)	191	2.08 (1.11–3.89)	0.005*
No	Current smoker	20 (31.7)	63	3.39 (2.20–5.20)	0.001*
Yes	Current smoker	16 (66.7)	24	6.62 (4.03–10.87)	0.001*
RERI	2.16 (0.67–3.64)				
AP	0.33 (0.18–0.47)				
SI	1.62 (1.22–2.15)				

Note: * represent the P value < 0.05.

## Discussion

Over the past decades, the influence of smoking or MetS on CRC has been widely investigated. Smoking has been demonstrated to have a significant association with tumorigenesis and poor CRC prognosis^8,13–15^. By studying 2548 CRC survivors, Baiyu Yang *et al*.^8^ indicated that pre- and post-diagnosis smoking status was associated with CRC-specific mortality. However, the association between MetS and CRC is currently unclear. As reported by some studies, MetS is a risk factor for CRC^16–19^. Moreover, several studies have demonstrated that CRC patients with MetS may have worse prognoses that CRC patients without MetS. Moreover, Jason R *et al*.^20^ found that male subjects with MetS had significantly higher risk of mortality from CRC than did male subjects without MetS. Two other studies also found similar results^21,22^. By studying 507 CRC patients, Shen Z *et al*.^23^ found that MetS was positively related to higher mortality and recurrence. You J *et al*.^11^ evaluated 1069 CRC patients and demonstrated that MetS was associated with an increased recurrence risk. However, some studies showed opposite results^12^. Nevertheless, until now, no study has investigated the additive effect of smoking and MetS on CRC prognoses. Thus, the present study aimed to investigate the potential individual and combined effect of smoking and MetS on the prognosis of CRC.

Initially, we found that MetS was independently associated with RFS and not OS, which is consistent with the findings of another study^11^. However, the present study employed the Chinese criteria for diagnosing MetS that are slightly different from the criteria in the abovementioned studies. Additionally, the participants were all Southeast Chinese males, who have unique dietary habits and heredity features. Previous studies have indicated that varying definitions of MetS and different races might influence clinical outcomes^24–26^ and thus partially explained the differences between the present study and others. Furthermore, the synergistic effect of MetS was determined using all of its components, which not only have complicated mechanisms but also are far from clear. The present study indicated that elevated BP and hyperglycemia have a negative effect on OS and RFS, while dyslipidemia exerts a positive effects on OS and RFS. Thus, we speculated that the protective effect of dyslipidemia on survival may have counteracted the effect of the two other risk factors and finally led to nonsignificant effects on OS. However, of those three significant components, elevated BP and hyperglycemia had dominant effects on RFS and resulted in poor RFS. The underlying mechanism should be further studied in the future. On the other hand, smoking status was significantly related to OS and RFS. In fact, there was no difference between “never” and “former” smokers regarding OS and RFS in Cox analyses, consistent with the results of another study^8^. Thus, we combined “former” and “never” smokers into the non-smoker group. Further evaluation on patients categorized into four groups based on their smoking and MetS status showed that smoking and MetS had significant additive effect on RFS.

At present, the potential mechanisms underlying the combined effect of smoking and MetS are not clearly understood. As indicated by previous studies, MetS may cause or promote CRC development and progression via various mechanisms, including dysregulation of growth signals, inflammatory cytokines, and vascular integrity factors^27^. Inflammation can contribute to the development, progression, and recurrence of tumors, including CRC^28,29^. More specifically, COX2, which involved in various inflammatory pathways, can promote the recurrence of adenomas and sporadic adenomatous polyps^30–32^. In addition, high expression of COX2 can induce CRC tumorigenesis and metastasis^33^. On the other hand, smoking can lead to inflammation through multiple mechanisms, including the regulation of secretion of tumor necrosis factor^34–36^. Smoking has been shown to induce inflammation associated with numerous diseases including CRC^37,38^. We observed an additive effect of smoking and MetS on recurrence. Hence, we hypothesized that inflammation may be a common mechanism underlying the pathophysiological process of recurrence induced by both MetS and smoking. However, the detailed mechanism should be further studied.

Previous studies have demonstrated that metformin may exert anti-cancer effects and reduce the incidence of various cancers, including CRC^39^. The association between metformin and CRC prognosis remains controversial. Based on our results, metformin appeared to play protective roles regarding both OS and RFS for CRC patients, consistent with the results of other studies^40,41^. However, some other studies did not support the protective association between metformin treatment and CRC prognosis^42^.

To the best of our knowledge, this is the first study examining the combined effect of smoking and MetS on CRC prognosis. Studies have indicated that metformin and statin treatment may play anti-cancer roles in various cancers^39,43^, but these effects have been neglected in numerous other studies. Thus, in the present study, we controlled for the confounding effects of metformin and statin and obtained more reasonable and reliable results. However, several limitations still exist. First, the observational study design does not provide evidence regarding the potential mechanism of the combined effect of smoking and MetS on recurrence. Second, the relatively short duration of follow-up, single center focus, and a small number of current smokers may weaken the results of the present study. Third, although the definition of MetS in the present study has been used in investigating various diseases^44–47^, it is not used worldwide. However, due to the unique hereditary and dietary features, this definition is suitable for the Chinese population. Finally, residual confounding cannot be avoided owing to the characteristics of observational studies. Indeed, we could not exclude the possibility of the effect of uncontrolled or inadequately measured confounders on the results. Therefore, a large multicenter prospective study with long-term follow-up should be implemented in the future.

## Conclusion

The present study indicates that smoking can influence both recurrence and mortality in CRC patients, while the presence of the MetS only affects recurrence, and these observations supplement the existing knowledge on the relationship among smoking, MetS, and prognosis in male CRC patients. Furthermore, this study demonstrates a additive effects of current smoking habits and MetS on recurrence risk, highlighting the need for extra postoperative management to reduce recurrence among Chinese male CRC patients who smoke and have MetS.

## Methods

### Study cohort

The present study enrolled participants who underwent primary surgical resection of CRC at our hospital from January 2010 to July 2016. None of the patients had distant metastasis at diagnosis. Patients who met the following conditions were excluded: 1) patients with history of any other cancer and 2) patients with familial adenomatous polyposis syndrome or hereditary nonpolyposis CRC. Clinicopathological and laboratory data of all patients were collected from electronic medical records and reviewed. The present study was approved by the Ethics Committee of the First Affiliated Hospital of Zhejiang University, and informed consent was obtained from every subject. The study procedure conformed to the Declaration of Helsinki and Strengthening the Reporting of Observational Studies in Epidemiology (STROBE) Statement^48^.

### Basic patient characteristics and laboratory measurements

A standard questionnaire with past medical history, smoking status, etc., was utilized to acquire the demographic information of patients. Trained nurses measured the height and weight of all patients. BMI was calculated as kg/m^2^, and BP was measured in a resting state with a standard mercury sphygmomanometer. Laboratory assays and measurements, including LDL cholesterol, HDL cholesterol, triglycerides (TGs) and other related blood parameters, were performed for all the participants.

All the CRC patients who were primarily diagnosed by coloscopy underwent surgical treatment along with adjuvant chemotherapy based on the National Comprehensive Cancer Network guidelines. Tumor staging of CRC was conducted according to the sixth edition of the American Joint Committee on Cancer Staging Manual. Other data regarding tumor location and histological differentiation were collected by pathological and colonoscopic sample analysis.

### Exposure assessment

Patients who had smoked at least 100 cigarettes and still smoked at the time of the interview were classified as current smokers. Patients who had smoked at least 100 cigarettes but had stopped for at least 2 years were classified as former smokers. Individuals without a history of cigarette smoking were classified as never smokers^49,50^.

We utilized the guideline proposed by the Diabetes Society of Chinese Medical Association (2004)^51^. Metabolic syndrome (MetS) was defined by the presence of three or more of the following components: (i) BMI ≥ 25 kg/m^2^; (ii) administration of anti-hypertensive drugs and (or) systolic blood pressure (SBP) ≥ 140 mmHg or diastolic blood pressure (DBP) ≥ 90 mmHg; (iii) TG ≥ 1.7 mmol/L and (or) HDL < 0.9 mmol/L (male) or < 1.0 mmol/L (female); and (iv) fasting plasma glucose ≥ 6.1 mmol/L or 2-h postprandial glucose ≥ 7.8 mmol/L.

### Follow-up and end point assessment

The frequencies of postoperative outpatient visits were as follows: every 3–6 months for 2 years, followed by every 6 months for a total of 5 years, and every 1 year thereafter. In each follow-up appointment, the smoking status was re-verified. The follow-up assessment included physical examination, tumor biomarker (serum CEA and CA19-9 levels) measurements, and chest and abdominal computed tomography (CT) and colonoscopy. Recurrence was identified based on abnormal imaging findings, cytology or biopsy examination. Overall survival (OS) was calculated from the date of surgery to the date of death or the date of last follow-up. Recurrence-free survival (RFS) was calculated as the time from the date of surgery to the date of recurrence or the date of last follow-up.

### Statistical analysis

Statistical analysis was performed with SPSS 19.0 (SPSS, Chicago, IL, USA). The data are presented as the mean ± standard deviation or percentages. Baseline characteristics were compared among six groups based on different smoking status and MetS status, and the chi-square test was used for categorical variables, and ANOVA was utilized for continuous variables. Furthermore, Kaplan-Meier survival curves with log-rank tests and Cox proportional hazards regression analysis were used to compare the OS and RFS rates. Variables with *P* < 0.05 in the univariate regression analysis were subjected to a multivariate Cox analysis. The individual components of MetS were adjusted by age, CEA level, stage, tumor location, tumor differentiation, metformin treatment, statin therapy and smoking status for the multivariate Cox analysis. All *P* values were two-sided, and *P* value < 0.05 was considered statistically significant.

The participants were divided into four groups according to their smoking and MetS status. The interaction between smoking and MetS on prognosis of CRC was evaluated by calculating the relative excess risk due to interaction (RERI), the attributable proportion (AP) due to interaction, and the synergy index (SI) based on the methods proposed by Andersson *et al*.^52^. When there was no additive interaction, RERI and AP were 0 or the SI was 1. RERI > 0, AP > 0, or SI > 1 indicated biological interaction.
